# Association between lactate-to-albumin ratio and all-cause mortality in patients with cerebrovascular disease: A retrospective cohort study

**DOI:** 10.1097/MD.0000000000044724

**Published:** 2025-09-26

**Authors:** Juan Xie, Xiaorui Zhang, Cheng Guo, Yang Li, Qingming Yang, Wenxin Fan, Kai Liu

**Affiliations:** a Department of Anesthesiology, Kunming Children’s Hospital & Children’s Hospital Affiliated to Kunming Medical University, Kunming, Yunnan, China; b Department of Pediatric, Affiliated Hospital of Kunming University of Science and Technology & The First People’s Hospital of Yunnan Province, Kunming, Yunnan, China; c Department of Comprehensive Pediatrics, Kunming Children’s Hospital & Children’s Hospital Affiliated to Kunming Medical University, Kunming, Yunnan, China.

**Keywords:** all-cause mortality, cerebrovascular disease, LAR, predictive performance

## Abstract

Intensive care unit (ICU) patients suffering from cerebrovascular disease are subject to a high mortality risk. The existing prognostic tools are characterized by their complexity and limited universality and timeliness. The lactate albumin ratio (LAR) has been shown to integrate tissue perfusion (lactate) and nutritional-inflammatory status (albumin), with the potential to serve as a simple, cost-effective risk stratification indicator. The present study evaluated the relationship between LAR and all-cause mortality during hospitalization and in the ICU among critically ill patients with cerebrovascular disease, and compared its predictive performance with that of commonly used inflammatory markers. We identified 873 ICU patients with cerebrovascular disease from MIMIC‑IV 3.0 and stratified them into LAR tertiles. Primary outcomes were in‑hospital and ICU all‑cause mortality. Associations between LAR and outcomes were assessed using Cox models, Kaplan–Meier curves, and restricted cubic splines; predictive performance was evaluated by receiver operating characteristic analysis. The Kaplan–Meier analysis revealed a significant association between an increase in LAR level and a decrease in survival rate. In the fully adjusted model, for every 1-unit increase in LAR, the risk of in-hospital death increased by 15% (HR = 1.15, 95% CI = 1.07–1.23, *P* < .001), and the risk of ICU death also increased by 15% (HR = 1.15, 95% CI = 1.06–1.24, *P* < .001). The restricted cubic spline analysis confirmed that LAR was approximately linearly positively correlated with the risk of death, and this relationship remained consistent across all subgroups. The area under the curve value of LAR was 0.68 (95% CI = 0.64–0.71), which was superior to traditional inflammatory indicators (platelet-to-lymphocyte ratio (PLR), lymphocyte ratio (NLR), systemic inflammation response index (SIRI), systemic inflammation index (SII)). The present study has demonstrated that LAR is an independent risk factor for in-hospital mortality in patients with cerebrovascular diseases, showing a nearly linear positive correlation. Its predictive performance has been demonstrated to exceed that of conventional inflammatory indicators (PLR, NLR, SIRI, SII). LAR’s simplicity of measurement and low cost suggests that it will serve as a supplementary tool for early risk stratification in the ICU.

## 1. Introduction

Cerebrovascular diseases refer to a general term for brain disorders caused by vascular lesions or blood flow disorders. The most common types include cerebral infarction, cerebral hemorrhage, subarachnoid hemorrhage and transient ischemic attack.^[1]^ Cerebrovascular diseases are the leading cause of both disability and death on a global scale, and represent the fifth most common cause of death in the United States.^[2]^ Patients suffering from cerebrovascular diseases who are admitted to the intensive care unit (ICU) have complex conditions and a poor prognosis, thus the early identification of high-risk patients is of particular importance.

The pathophysiological mechanisms of cerebrovascular diseases are closely related to tissue ischemia, energy metabolism imbalance, and inflammatory responses. Lactate and albumin serve as 2 key biomarkers that play important roles in disease progression and prognosis assessment.^[3,4]^ Lactate is the principal product of anaerobic metabolism in the context of cerebral tissue ischemia and hypoxia. Post-cerebrovascular events are characterized by circulatory disturbances, both locally and systemically. These disturbances result in insufficient cerebral perfusion, which, in turn, leads to enhanced glycolysis and impaired aerobic metabolism. The consequence of these processes is the promotion of massive lactate production.^[5]^ Research has demonstrated that serum lactate levels in patients with cerebrovascular diseases are significantly associated with the degree of neurological deficit, clinical outcomes at discharge, and long-term mortality.^[6]^ In patients suffering from severe cerebral infarction and cerebral hemorrhage, persistently elevated blood lactate levels are indicative of inadequate tissue perfusion and severe metabolic disorders. These are not limited to the local brain tissue but represent manifestations of systemic multi-organ dysfunction.^[7]^

Meanwhile, albumin, an important carrier protein and antioxidant in the body, also plays a crucial role in cerebrovascular diseases. During the acute phase of cerebrovascular disease, patients frequently exhibit diminished serum albumin levels due to inflammatory responses, augmented vascular permeability, and deteriorating nutritional status.^[8]^ Hypoalbuminemia has been demonstrated to reflect poor nutritional status in patients, and is also closely associated with reduced oxidative stress capacity, endothelial dysfunction, and enhanced systemic inflammatory responses.^[9]^ Albumin exerts a protective effect through mechanisms such as scavenging free radicals, maintaining vascular endothelial integrity, and inhibiting the release of inflammatory factors. A decline in its levels directly affects the prognosis of patients with cerebrovascular diseases.^[10]^ A plethora of clinical studies have determined that albumin levels at admission serve as independent predictors of in-hospital mortality and long-term functional recovery in stroke patients. It has been observed that a decrease of 1 g/dL in albumin concentration results in an approximate increase in the risk of death by 1.5 to 2.0 times.^[11]^

In recent years, the lactate-albumin ratio (LAR), which combines these 2 indicators, has gradually gained attention. The integration of lactate, a reflection of tissue perfusion and metabolic status, with albumin, a reflection of inflammatory and nutritional status, by LAR provides a more comprehensive reflection of the patient’s pathophysiological state. In critical illnesses such as sepsis, trauma, and myocardial infarction, LAR has been proven to have good prognostic predictive value.^[12,13]^ From a pathophysiological perspective, the synergistic effects of elevated lactate and decreased albumin may exist in cerebrovascular diseases. The acidotic environment caused by elevated lactate activates the NF-κB pathway, promoting the release of inflammatory factors.^[14]^ Meanwhile, the reduced free radical scavenging capacity due to decreased albumin further exacerbates oxidative stress.^[15]^ Consequently, the combination of these factors results in a continuous, self-reinforcing pathological cycle that damages vascular endothelial function, leading to microcirculatory perfusion disorders, organ hypoxia, immune dysfunction, and a significant increase in the risk of multiple organ dysfunction and death.^[16]^

The present study has been designed to explore the correlation between LAR and all-cause mortality in patients suffering from cerebrovascular diseases. In addition, the study will evaluate LAR’s performance in predicting all-cause mortality in this group of patients. The ultimate aim of the study is to provide a more convenient and effective risk assessment tool for clinical practice.

## 2. Methods

### 2.1. Data collection

The data utilized in this study are drawn from MIMIC-IV, an electronic health record dataset encompassing over 50,000 patients admitted to the ICU at Boston Institute for Biomedical Research in Massachusetts from 2008 to 2019. The Institutional Review Board of BIDMC approved the exemption from informed consent and the sharing of research resources. The author (XRZ) was granted access to the database with certificate number 69398148.

### 2.2. Inclusion and exclusion criteria for the study population

**Figure 1.**
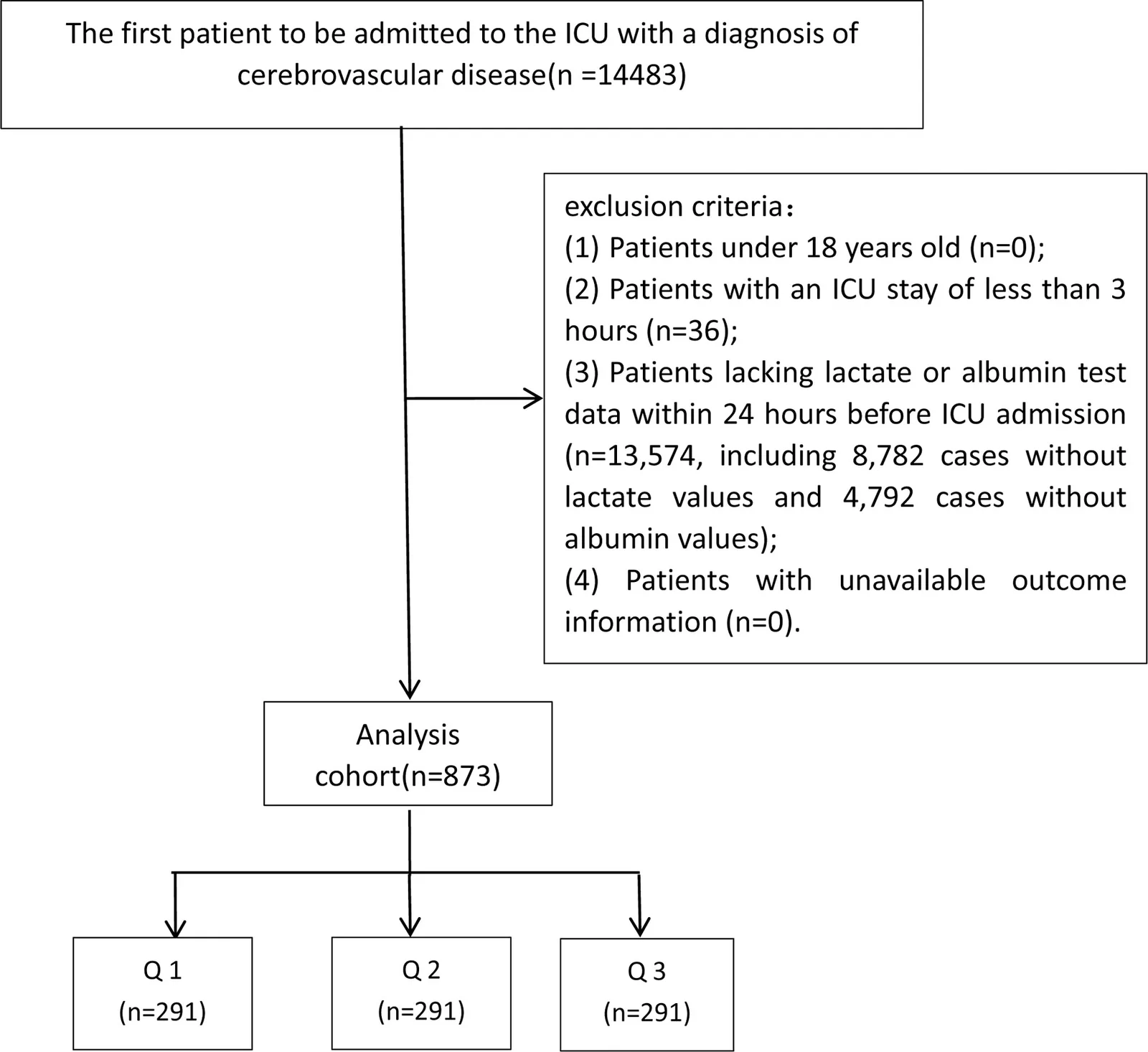
Flowchart of select study population from the MIMIC-IV database.

This study initially included 14,483 patients who were admitted to the ICU for the first time and diagnosed with cerebrovascular disease, based on international classification of diseases codes. To ensure data quality and research feasibility, we established the following exclusion criteria (Fig. 1): Patients under 18 years old (n = 0); Patients with an ICU stay of <3 hours (n = 36); Patients lacking lactate or albumin test data within 24 hours before ICU admission (n = 13,574, including 8782 cases without lactate values and 4792 cases without albumin values); and Patients with unavailable outcome information (n = 0). After rigorous screening, 873 patients were ultimately included in the study analysis, and they were divided into 3 groups based on the median LAR, with 291 patients in each group.

### 2.3. Clinical outcomes

The primary endpoints of this study were all-cause mortality during hospitalization and all-cause mortality in the ICU.

### 2.4. Statistical analysis

Continuous variables are represented by mean ± standard deviation or median (interquartile range), while categorical variables are presented as frequency (percentage). The relationship between LAR and survival time was assessed using Kaplan–Meier curves. Cox proportional hazards regression models were employed to evaluate the association between LAR and hospitalization and ICU all-cause mortality, with 3 models constructed: Model 1 was unadjusted; Model 2 was adjusted for age, sex, and race; and Model 3 was further adjusted for comorbidities and laboratory tests, among other confounding factors. Restricted cubic splines (RCS) were utilized to investigate the approximate linear relationship between LAR and mortality risk. Subgroup analysis evaluated the predictive stability of LAR in different populations through interaction effect testing. Receiver operating characteristic (ROC) curve analysis was employed to assess the predictive performance of LAR and traditional inflammatory markers (platelet-to-lymphocyte ratio (PLR), lymphocyte ratio (NLR), systemic inflammation response index (SIRI), systemic inflammation index (SII)) in predicting mortality in patients with cerebrovascular disease. All analyses were conducted using R software (version 4.0.3), and a two-tailed *P* < .05 was considered statistically significant.

## 3. Results

### 3.1. Population baseline characteristics

A total of 873 patients with cerebrovascular diseases were included in this study. The patients were divided into 3 groups based on the tertiles of LAR: Q1 group, Q2 group and Q3 group. The mean LAR was 1.30 ± 1.48. Concurrently, the LAR-induced elevation in heart rate and respiratory rate was accompanied by a significant decrease in blood pressure (all *P* < .0001). Laboratory indicators demonstrated that elevated LAR values were associated with diminished albumin levels (from 3.31 ± 0.56 in Q1 to 2.80 ± 0.62 in Q3, *P* < .0001) and a considerable augmentation in white blood cell count (from 11.43 ± 4.96 in Q1 to 16.51 ± 8.75 in Q3, *P* < .0001). The severity scores (sequential organ failure assessment (SOFA), SAPSII, acute physiology and chronic health evaluation III, and oxford acute severity of illness score) all demonstrated a significant increase with higher LAR levels (all *P* < .0001). A clear positive correlation was evident between LAR levels and mortality, with three-median hospital mortality rates of 18.21%, 30.24%, and 37.80% (*P* < .0001) and ICU mortality rates of 13.06%, 20.96%, and 30.58% (*P* < .0001). However, no statistically significant differences were observed in hospital or ICU stay duration among the study groups (*P* > .05) (Table 1).

**Table 1:** Baseline characteristics of the LAR tertile population.

Variable	Total (n = 873)	Q1 (n = 291)	Q2 (n = 291)	Q3 (n = 291)	*P* value
Sex					
Female	381 (43.64)	127 (43.64)	130 (44.67)	124 (42.61)	.88
Male	492 (56.36)	164 (56.36)	161 (55.33)	167 (57.39)	
Age	67.79 (57.33, 78.16)	67.82 (56.89, 77.22)	67.29 (56.82, 78.33)	68.08 (58.65, 78.87)	.78
Race					
Black	122 (13.97)	36 (12.37)	39 (13.40)	47 (16.15)	.09
Other	269 (30.81)	97 (33.33)	75 (25.77)	97 (33.33)	
White	482 (55.21)	158 (54.30)	177 (60.82)	147 (50.52)	
HR	86.91 ± 16.16	82.26 ± 14.50	87.26 ± 14.95	91.21 ± 17.64	<.0001
SBP	118.67 ± 16.15	123.12 ± 15.86	118.64 ± 16.56	114.25 ± 14.79	<.0001
DBP	63.91 ± 10.82	65.53 ± 11.29	63.79 ± 10.60	62.41 ± 10.35	<.01
MBP	79.74 ± 10.63	82.04 ± 11.17	79.42 ± 10.46	77.76 ± 9.81	<.0001
RR	20.27 ± 4.34	19.24 ± 3.84	20.23 ± 4.46	21.36 ± 4.43	<.0001
Spo2	97.27 ± 2.37	97.40 ± 1.98	97.34 ± 2.01	97.06 ± 2.97	.17
Myocardial_infarction					
No	671 (76.86)	232 (79.73)	222 (76.29)	217 (74.57)	.32
Yes	202 (23.14)	59 (20.27)	69 (23.71)	74 (25.43)	
Heart_failure					
No	559 (64.03)	192 (65.98)	182 (62.54)	185 (63.57)	.68
Yes	314 (35.97)	99 (34.02)	109 (37.46)	106 (36.43)	
Atrial_fibrillation					
No	558 (63.92)	195 (67.01)	175 (60.14)	188 (64.60)	.22
Yes	315 (36.08)	96 (32.99)	116 (39.86)	103 (35.40)	
Diabetes					
No	526 (60.25)	189 (64.95)	167 (57.39)	170 (58.42)	.13
Yes	347 (39.75)	102 (35.05)	124 (42.61)	121 (41.58)	
Hypertension					
No	511 (58.53)	166 (57.04)	170 (58.42)	175 (60.14)	.75
Yes	362 (41.47)	125 (42.96)	121 (41.58)	116 (39.86)	
Sepsis					
No	552 (63.23)	227 (78.01)	168 (57.73)	157 (53.95)	<.0001
Yes	321 (36.77)	64 (21.99)	123 (42.27)	134 (46.05)	
COPD					
No	679 (77.78)	227 (78.01)	221 (75.95)	231 (79.38)	.60
Yes	194 (22.22)	64 (21.99)	70 (24.05)	60 (20.62)	
Renal_disease					
No	620 (71.02)	211 (72.51)	206 (70.79)	203 (69.76)	.76
Yes	253 (28.98)	80 (27.49)	85 (29.21)	88 (30.24)	
PLT	197.65 ± 102.87	207.98 ± 102.62	201.45 ± 104.65	183.52 ± 100.07	.01
RBC	3.60 ± 0.72	3.62 ± 0.70	3.54 ± 0.73	3.64 ± 0.73	.18
WBC	14.03 ± 7.36	11.43 ± 4.96	14.16 ± 6.98	16.51 ± 8.75	<.0001
Bun	34.35 ± 26.42	29.63 ± 26.65	34.26 ± 23.97	39.16 ± 27.73	<.0001
sCr	2.00 ± 2.06	1.83 ± 2.55	1.93 ± 1.78	2.23 ± 1.73	.06
SOFA	6.97 ± 4.02	5.15 ± 3.09	6.66 ± 3.90	9.09 ± 3.98	<.0001
Sapsii	43.96 ± 15.29	37.65 ± 12.17	42.23 ± 13.65	51.99 ± 16.14	<.0001
Aps3	57.07 ± 25.05	46.23 ± 18.35	54.31 ± 21.18	70.68 ± 28.12	<.0001
Oasis	36.61 ± 8.34	34.04 ± 7.50	35.95 ± 7.94	39.85 ± 8.51	<.0001
PLR	23.93 ± 26.18	22.58 ± 20.30	25.29 ± 29.53	23.94 ± 27.85	.18
NLR	1.44 ± 2.37	1.14 ± 1.44	1.47 ± 2.52	1.72 ± 2.89	<.0001
SIRI	1.41 ± 4.34	0.89 ± 1.40	1.66 ± 5.65	1.69 ± 4.76	<.001
SII	305.84 ± 518.82	254.35 ± 381.63	331.32 ± 642.86	332.66 ± 497.89	<.01
los_hospital_day	17.75 ± 21.31	18.15 ± 26.36	17.52 ± 19.06	17.58 ± 17.53	.93
los_icu_day	7.57 ± 10.33	7.27 ± 11.47	7.65 ± 10.01	7.80 ± 9.43	.82
death_hosp_yn					
Alive	622 (71.25)	238 (81.79)	203 (69.76)	181 (62.20)	<.0001
Death	251 (28.75)	53 (18.21)	88 (30.24)	110 (37.80)	
death_icu_yn					
Alive	685 (78.47)	253 (86.94)	230 (79.04)	202 (69.42)	<.0001
Death	188 (21.53)	38 (13.06)	61 (20.96)	89 (30.58)	

APS3 = acute physiology and chronic health evaluation III, BUN = blood urea nitrogen, COPD = chronic obstructive pulmonary disease, DBP = diastolic blood pressure, HR = heart rate, LAR = lactate-albumin ratio, MBP = mean arterial pressure, NLR = lymphocyte ratio, OASIS = oxford acute severity of illness score, PLR = platelet-to-lymphocyte ratio, PLT = platelets or platelet count, RBC = red blood cell count, RR = respiratory rate, SAPS-II = acute physiology score II, SBP = systolic blood pressure, sCr = serum creatinine, SII = systemic inflammation index, SIRI = systemic inflammation response index, SOFA = sequential organ failure assessment, SpO2 = peripheral oxygen saturation, WBC = white blood cell count.

### 3.2. LAR and clinical outcomes

**Figure 2.**
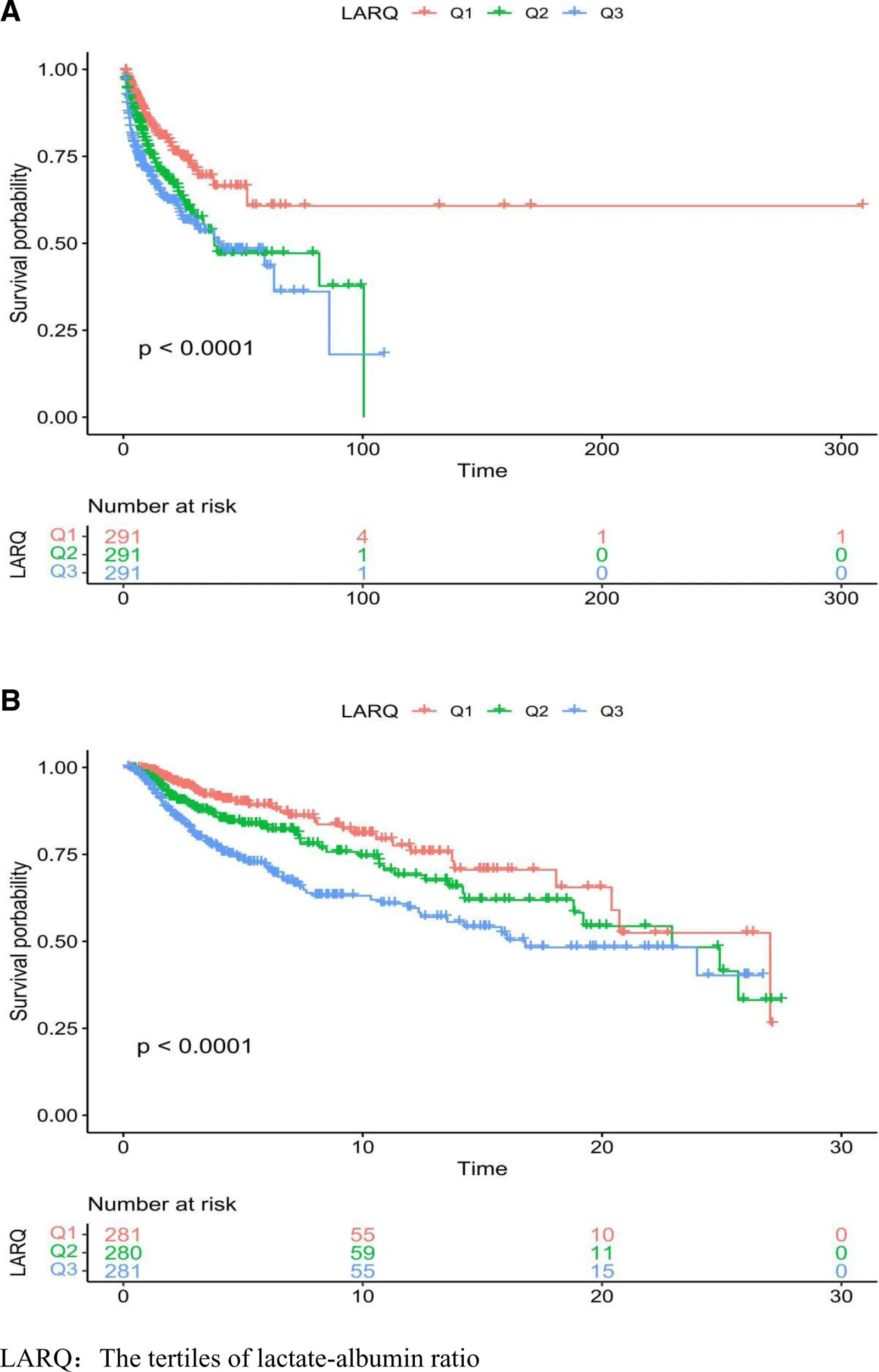
Kaplan–Meier survival analysis curves for all-cause mortality. (A) In-hospital mortality. (B) ICU mortality. ICU = intensive care unit.

The Kaplan–Meier survival curve clearly shows (Fig. 2) that the LAR level increased from Q1 to Q3, leading to a significant decrease in both hospitalization and ICU survival rates (both *P* < .0001). The results of the multivariate Cox regression analysis (Table 2) indicate that, in the fully adjusted model (Model 2), for each unit increase in LAR as a continuous variable, the risk of hospitalization-related death increases by 15% (HR = 1.15, 95% CI: 1.07–1.23, *P* < .001), and the risk of ICU-related death also increases by 15% (HR = 1.15, 95% CI: 1.06–1.24, *P* < .001). Using the lowest LAR group (Q1) as the reference, after adjusting for age, gender, and race (Model 1), the hospitalization-related death risks in Q2 and Q3 groups increased by 76% (*P* = .001) and 121% (*P* < .0001), respectively. After further adjustment for clinical factors such as myocardial infarction and heart failure, as well as laboratory indicators (Model 2), the hospitalization-related death risks in Q2 and Q3 groups still increased by 51% (*P* = .02) and 67% (*P* = .01), respectively. For ICU-related deaths, the risk in Q3 group increased by 69% (*P* = .01) after full adjustment, while the risk in Q2 group increased by 44%, but not statistically significant (*P* = .09). The trend analysis showed that with the increase of LAR level, the risk of hospitalization and ICU death increased significantly linearly (both *P* < .05) regardless of the adjustment model used, suggesting that LAR could be an independent risk factor for predicting the prognosis of patients with cerebrovascular disease.

**Table 2:** Cox proportional hazard models for in-hospital and in-ICU all-cause mortality.

Character	Crude model		Model 1		Model 2	
	95% CI	*P*	95% CI	*P*	95% CI	*P*
death_hosp~LAR	1.2 (1.13, 1.27)	<.0001	1.21 (1.14, 1.29)	<.0001	1.15 (1.07, 1.23)	<.001
death_hosp~LARQ						
Q1	Ref		Ref		Ref	
Q2	1.69 (1.21, 2.38)	.002	1.76 (1.25, 2.47)	.001	1.51 (1.06, 2.16)	.02
Q3	2.16 (1.55, 3.00)	<.0001	2.21 (1.59, 3.06)	<.0001	1.67 (1.17, 2.39)	.01
*P* for trend		<.0001		<.0001		.01
death_icu~LAR	1.2 (1.12, 1.28)	<.0001	1.2 (1.13, 1.29)	<.0001	1.15 (1.06, 1.24)	<.001
death_icu~LARQ						
Q1	Ref		Ref		Ref	
Q2	1.53 (1.02, 2.29)	.04	1.57 (1.04, 2.35)	.03	1.44 (0.94, 2.19)	.09
Q3	2.18 (1.49, 3.18)	<.0001	2.2 (1.50, 3.21)	<.0001	1.69 (1.11, 2.57)	.01
*P* for trend		<.0001		<.0001		.02

Crudel model: LAR. Model 1: LAR, age, sex, race; Model 2: LAR, age, sex, race, Myocardial_infarction, heart_failure, Atrial_fibrillation, diabetes, Hypertension, sepsis, COPD, Renal_disease, HR, mbp, RR, spo2, RBC, WBC, bun, sCr.

ICU = intensive care unit, LAR = lactate-albumin ratio.

**Figure 3.**
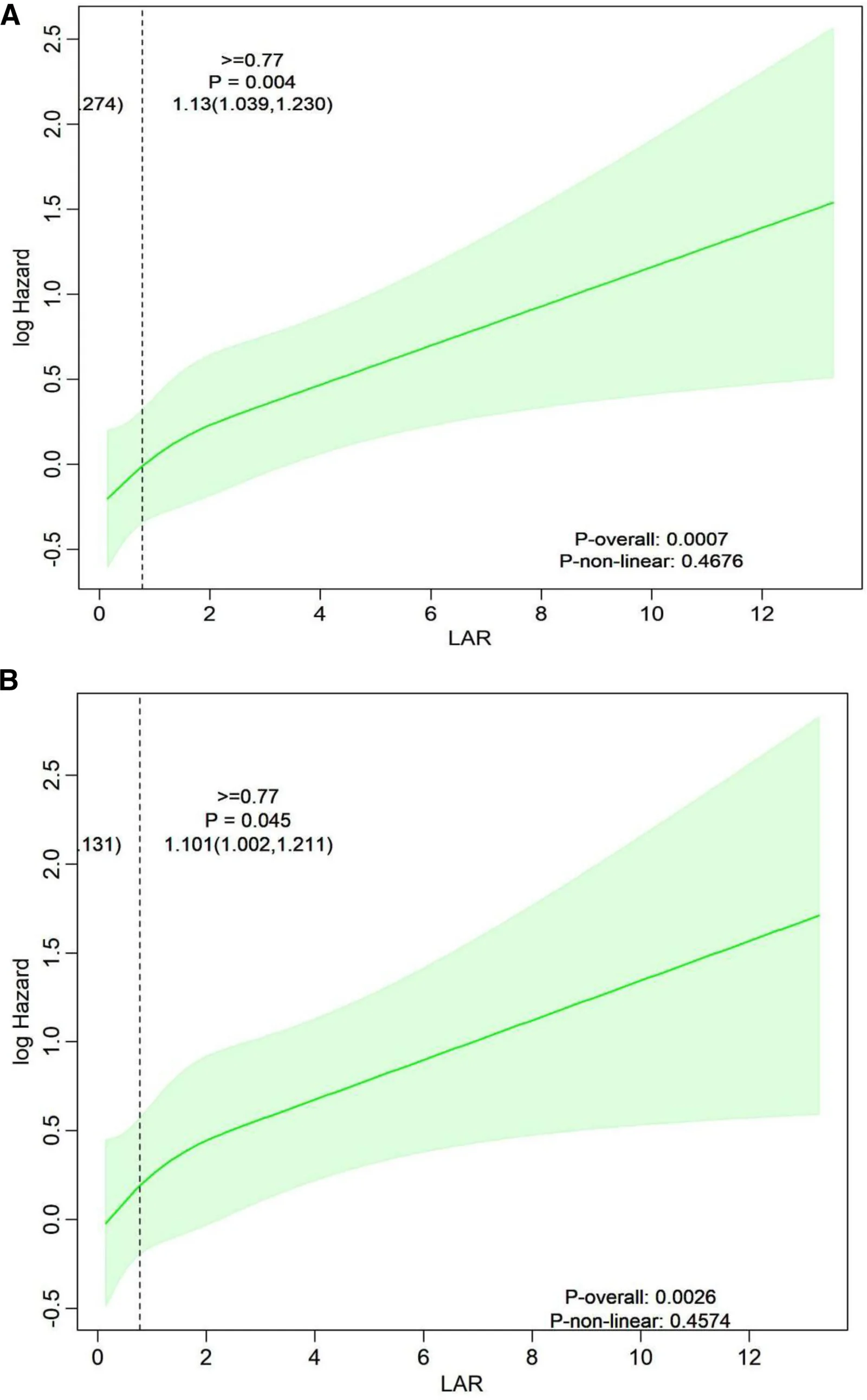
RCS analysis of LAR with all-cause mortality. (A) In-hospital mortality. (B) ICU mortality. LAR = lactate-albumin ratio, RCS = restricted cubic splines.

Furthermore, RCS were utilized to analyze the dose-response relationship between LAR and patient mortality risk (Fig. 3). The findings demonstrate a statistically significant positive correlation between LAR values and both the all-cause mortality rate in hospitalization (Fig. 3A) and the all-cause mortality rate in ICU (Fig. 3B). The *P*-values for the overall analysis are .0007 for hospitalization and .0026 for ICU. The trend in the graphs indicates that as the LAR value increases from 0 to approximately 2, the mortality risk rises rapidly; although the growth rate slows down slightly, it continues to increase. The non-linear tests for both figures were not significant (hospitalization: *P*-non-linear = .4676; ICU: *P*-non-linear = .4574), suggesting that the relationship between LAR and mortality risk is essentially linear.

### 3.3. Subgroup analysis

**Figure 4.**
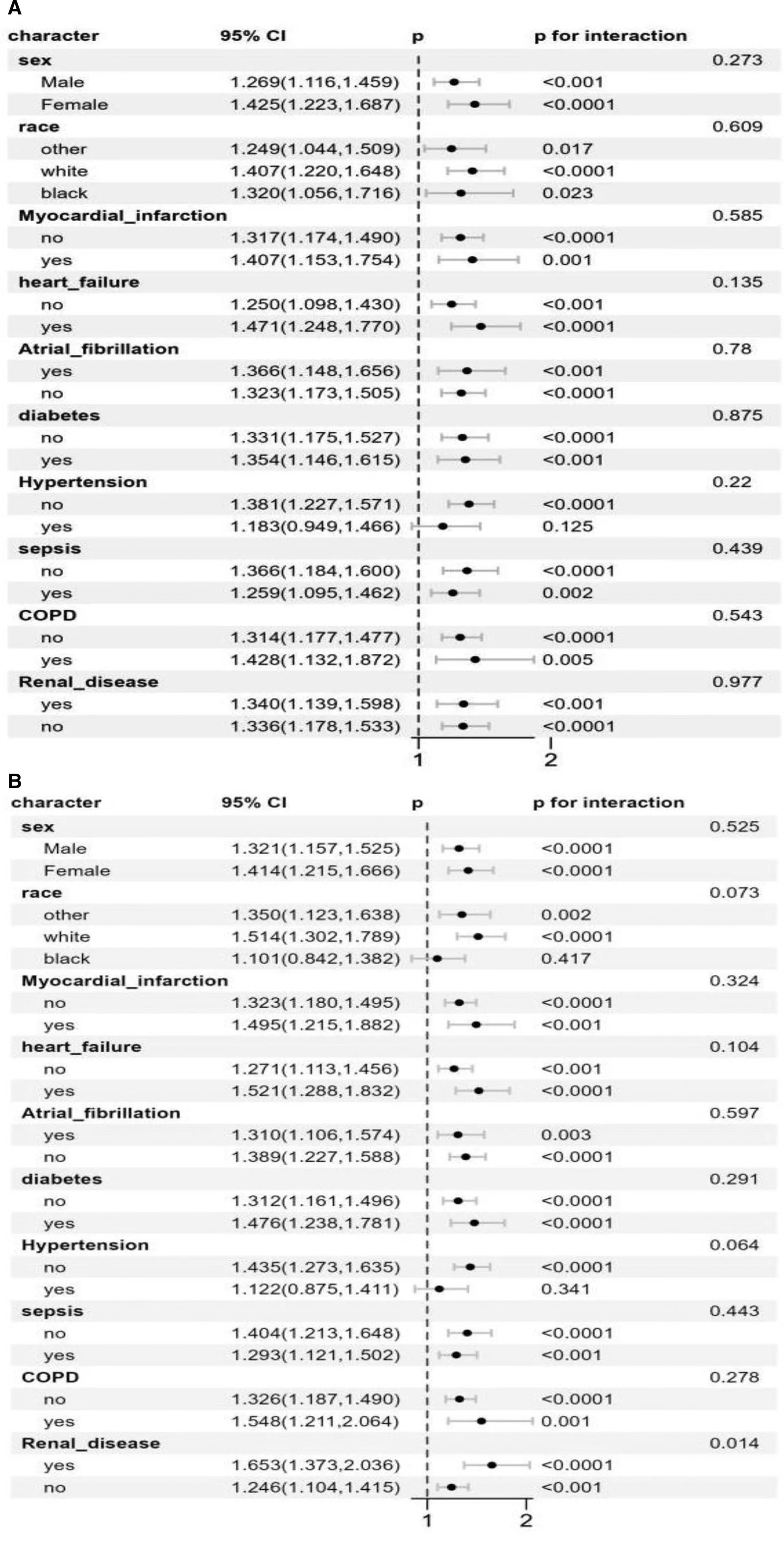
Subgroup forest plot for all-cause mortality. (A) In-hospital mortality. (B) ICU mortality. ICU = intensive care unit.

The forest plot analysis (Fig. 4) demonstrates that LAR exhibits a statistically significant positive correlation with the risk of hospitalization and death in nearly all subgroups, encompassing patients of diverse genders, races, and those with a range of underlying conditions (most *P* < .01). The subgroup interaction analysis indicates that the impact of LAR on the risk of hospitalization and death is not significantly different across subgroups (all *P* for interaction > .05), thus confirming the robustness of LAR as a predictive factor. In the context of ICU mortality risk, LAR has been demonstrated to exhibit significant predictive value in the majority of subgroups. A notable interaction was identified between the renal disease subgroup and LAR (*P* for interaction = .014), indicating that LAR may exhibit heightened sensitivity in predicting ICU mortality in patients with kidney disease (HR = 1.653, 95% CI: 1.373–2.036), while its predictive efficacy in patients without kidney disease is comparatively modest (HR = 1.246, 95% CI: 1.104–1.415).

### 3.4. The predictive performance of LAR on all-cause mortality in hospitalized patients with cerebrovascular disease

**Figure 5.**
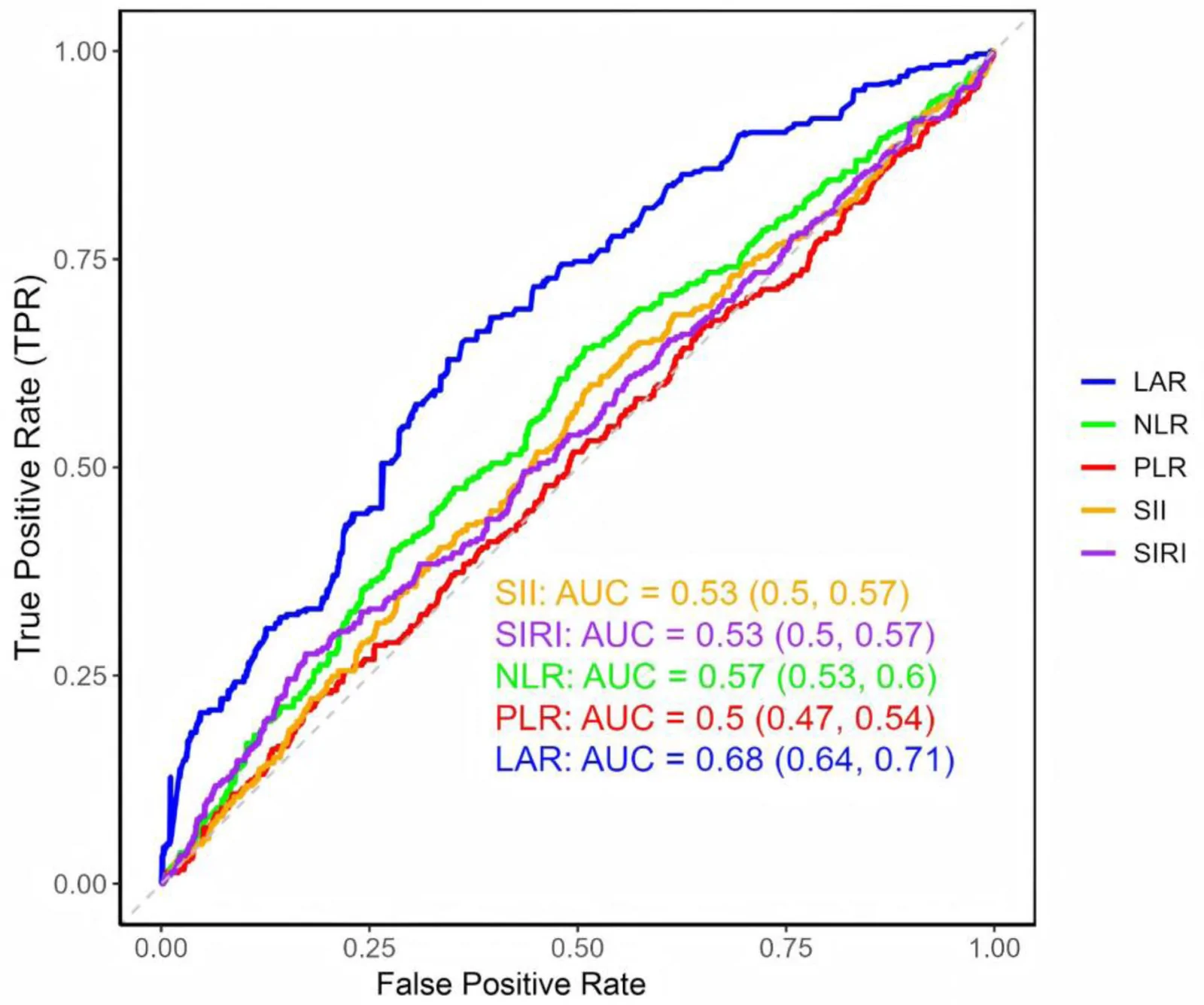
The ROC curve showed the predictive ability of LAR, NLR, PLR, SII and SIRI for all-cause mortality in patients with cerebrovascular disease. LAR = lactate-albumin ratio, NLR = lymphocyte ratio, PLR = platelet-to-lymphocyte ratio, SII = systemic inflammation index, SIRI = systemic inflammation response index.

Figure 5 compares the ROC curves of the LAR, NLR, PLR, SII, and SIRI in predicting mortality risk. The area under the curve (AUC) values for each indicator are as follows: The AUC of the LAR is 0.68 (95% CI: 0.64–0.71), indicating high predictive power; the AUC of the NLR is 0.57 (95% CI: 0.53–0.6), suggesting moderate predictive ability; the AUC of the PLR is 0.50 (95% CI: 0.47–0.54), indicating poor accuracy; the AUC of both the SII and the SIRI is 0.53, indicating limited clinical utility.

## 4. Discussion

The present study, based on the large public cohort of MIMIC-IV 3.0, systematically evaluated the predictive value of LAR for the risk of hospitalization and ICU mortality in critically ill patients with cerebrovascular disease. The present study addresses the long-standing neglect of the dual information integration index of “perfusion metabolism and inflammation-nutrition” in current prognosis studies. The findings indicate a close linear relationship between LAR and mortality risk, as evidenced by an AUC of 0.68, which exceeds the performance of widely applied inflammatory indicators (NLR, PLR, SIRI, SII). This finding indicates that LAR, as a straightforward metric integrating tissue perfusion and inflammation/nutrition data, has the potential to enhance existing ICU scoring systems. Furthermore, the model exhibits augmented predictive capability within the renal insufficiency subgroup, signifying its capacity to discern imbalances in the multi-organ metabolic-inflammation chain with heightened sensitivity. LAR can be obtained through a single routine blood gas and biochemical test, which renders it both cost-effective and simple to perform. In ICU with limited resources, LAR can serve as an auxiliary tool to complex scoring systems such as the SOFA and the simplified acute physiology score II, helping physicians to swiftly identify high-risk patients early in the ICU admission process. This approach has the potential to optimize the allocation of beds and monitoring resources, thereby providing a feasible reference point for the development of precise future treatments targeting high-lactate-low-albumin phenotypes. Such treatments may include enhanced haemodynamic management, nutritional support, and inflammation control.

The present research findings are highly consistent with those of previous studies on the clinical predictive value of inflammatory markers. As demonstrated by Liu et al,^[17]^ a comprehensive set of inflammatory markers has been shown to significantly predict poor outcomes in critically ill patients, and the present study further supports this key finding. As Sih et al also confirmed, the use of LAR has been demonstrated to be an effective predictor of the 28-day mortality rate in critically ill sepsis patients.^[13]^ Zhou et al discovered that LAR functions as an independent predictor of mortality in patients diagnosed with fever and thrombocytopenia syndrome.^[18]^ This may be due to elevated LAR, which is indicative of lactic acid accumulation resulting from inadequate tissue perfusion and the depletion of albumin under systemic inflammation. These factors mutually reinforce each other, collectively indicating a poor prognosis. In cases where tissue perfusion is inadequate, the accumulation of lactic acid is indicative of metabolic disorders and hypoxia.^[19]^ Conversely, decreased albumin has been shown to be a marker of weakened antioxidant capacity and enhanced systemic inflammation.^[20]^ At the molecular level, the lactic acid-induced acidosis environment activates the NF-κB pathway, promoting the release of inflammatory factors,^[14]^ and works synergistically with the free radical scavenging impairment caused by decreased albumin,^[15]^ together damaging endothelial function, exacerbating oxidative stress, forming a continuously self-reinforcing pathological cycle, ultimately leading to microcirculatory perfusion disorders, organ oxygen deficiency, immune dysfunction, and significantly increasing the risk of multiple organ dysfunction and death.^[16]^

In contrast to the findings of previous studies, the present investigation is pioneering in its evaluation of the predictive value of LAR in critically ill patients with diverse cerebrovascular diseases. A substantial body of research has been dedicated to the study of biomarkers of cerebrovascular disease, with previous studies focusing primarily on inflammatory markers or specific neurological injury markers.^[21,22]^ However, LAR, which integrates tissue perfusion and systemic inflammatory responses, may provide a more comprehensive reflection of the pathophysiological state of patients with cerebrovascular disease.^[23]^ Furthermore, the present findings suggest that LAR enhances the prediction of ICU mortality risk in patients with kidney disease.^[24]^ This phenomenon may be attributed to the synergistic effects of multiple pathophysiological mechanisms. Firstly, patients with renal insufficiency have significantly reduced lactate clearance, as the kidneys not only consume about 30% of circulating lactate through gluconeogenesis but also play a crucial role in direct lactate excretion.^[25,26]^ Secondly, there is a bidirectional causal relationship between kidney disease and hypoalbuminemia.^[23]^ As indicated by the extant literature, nephrotic syndrome leads to increased albuminuria and loss, while hypoalbuminemia has been demonstrated to exacerbate glomerular filtration membrane damage, creating a vicious cycle.^[27,28]^ Thirdly, patients with renal insufficiency often experience acid-base imbalance, which affects lactate metabolism and exacerbates inflammation. Oxidative stress and endothelial dysfunction are more severe in uremic conditions.^[29,30]^ Fourth, abnormal vascular regulation and the activation of the renin-angiotensin system can further worsen tissue perfusion, leading to more severe hypoxia and increased lactic acid production.^[31,32]^ Therefore, in patients with kidney disease, LAR may more sensitively reflect the severity of the disease and prognosis.^[33,34]^ The results indicate that LAR has significant clinical utility: it integrates indicators reflecting 2 critical pathophysiological processes (tissue perfusion and systemic inflammation/nutritional status), is easy to test, cost-effective, and particularly suitable for medical settings with limited resources.

The present study is subject to several limitations. Firstly, as a retrospective study based on the MIMIC-IV database, all patients are from a single-center population in the United States, and the results may not be directly applicable to patient populations in other regions or healthcare systems. Secondly, due to the inherent limitations of public databases, even after adjusting for multiple known confounding factors, there may still be unmeasured confounders that were not included in the analysis, such as lifestyle, dietary habits, and previous medications. Thirdly, while incorporating LAR into existing scoring systems statistically improved predictive power, the improvement was relatively limited, and the final AUC did not reach an ideal level (≥0.75). This finding indicates that, while LAR enhances risk stratification at the population level, its incremental clinical benefit for individual patients may be constrained. Fourthly, the impact of dynamic changes in LAR on prognosis was not assessed, which may have more clinical value than a single measurement. In conclusion, given the observational nature of the study, it is not possible to establish a clear causal relationship between LAR and mortality risk. It is therefore recommended that future studies with larger samples are conducted in order to confirm the findings of the present study.

## 5. Conclusion

The present study found that LAR was an independent risk factor for mortality in patients with cerebrovascular disease, and the 2 showed a nearly linear positive correlation. Furthermore, LAR demonstrated a capacity for prognostication, with the potential to enhance the precision of identifying patients who are at elevated risk of unfavorable outcomes.

## Acknowledgments

We acknowledge the contributions of the MIMIC-IV program registry for their work in creating and maintaining the MIMIC IV database.

## Author contributions

**Investigation:** Qingming Yang, Wenxin Fan.

**Methodology:** Juan Xie, Cheng Guo.

**Project administration:** Kai Liu.

**Resources:** Yang Li.

**Supervision:** Cheng Guo.

**Writing – original draft:** Xiaorui Zhang.

**Writing – review & editing:** Kai Liu.
